# Tailoring the Li^+^ Intercalation Energy of Carbon Nanocage Anodes Via Atomic Al‐Doping for High‐Performance Lithium‐Ion Batteries

**DOI:** 10.1002/smll.202406309

**Published:** 2024-10-02

**Authors:** Xingmiao Yu, Jianfei Xiang, Qitao Shi, Luwen Li, Jiaqi Wang, Xiangqi Liu, Cheng Zhang, Zhipeng Wang, Junjin Zhang, Huimin Hu, Alicja Bachmatiuk, Barbara Trzebicka, Jin Chen, Tianxiao Guo, Yanbin Shen, Jinho Choi, Cheng Huang, Mark H. Rümmeli

**Affiliations:** ^1^ Soochow Institute for Energy and Materials Innovation College of Energy Key Laboratory of Advanced Carbon Materials and Wearable Energy Technologies of Jiangsu Province Key Laboratory of Core Technology of High Specific Energy Battery and Key Materials for Petroleum and Chemical Industry Soochow University Suzhou 215006 P. R. China; ^2^ i‐Lab, CAS Center for Excellence in Nanoscience Suzhou Institute of Nano‐Tech and Nano‐Bionics (SINANO) Chinese Academy of Sciences (CAS) Suzhou 215123 P. R. China; ^3^ Institute for Complex Materials IFW Dresden 20 Helmholtz Strasse 01069 Dresden Germany; ^4^ LUKASIEWICZ Research Network PORT Polish Center for Technology Development Stablowicka 147 Wroclaw 54‐066 Poland; ^5^ Key Laboratory of Core Technology of High Specific Energy Battery and Key Materials for Petroleum and Chemical Industry Soochow University Suzhou 215006 P. R. China; ^6^ Physics and Energy Department College of Optical and Electronic Information Suzhou City University Suzhou 215104 P. R. China; ^7^ Jiangsu Key Laboratory of Advanced Negative Carbon Technologies Soochow University Suzhou 215123 P. R. China; ^8^ Centre of Polymer and Carbon Materials Polish Academy of Sciences M. Curie‐Sklodowskiej 34 Zabrze 41‐819 Poland; ^9^ Institute of Environmental Technology (IET), Centre for Energy and Environmental Technologies (CEET) VSB—Technical University of Ostrava 17 Listopadu 15 Ostrava 708 33 Czech Republic

**Keywords:** anode, atomic Al‐doping, carbon nanocage, fast‐charging

## Abstract

Graphitic carbon materials are widely used in lithium‐ion batteries (LIBs) due to their stability and high conductivity. However, graphite anodes have low specific capacity and degrade over time, limiting their application. To meet advanced energy storage needs, high‐performance graphitic carbon materials are required. Enhancing the electrochemical performance of carbon materials can be achieved through boron and nitrogen doping and incorporating 3D structures such as carbon nanocages (CNCs). In this study, aluminum (Al) is introduced into CNC lattices via chemical vapor deposition (CVD). The hollow structure of CNCs enables fast electrolyte penetration. Density functional theory (DFT) calculations show that Al doping lowers the intercalation energy of Li^+^. The Al–boron (B)–nitrogen (N‐doped CNC (AlBN‐CNC) anode demonstrates an ultrahigh rate capacity (≈300 mAh g^−1^ at 10 A g^−1^) and a prolonged fast‐charging lifespan (862.82 mAh g^−1^ at 5 A g^−1^ after 1000 cycles), surpassing the N‐doped or BN‐doped CNCs. Al doping improves charging kinetics and structural stability. Surprisingly, AlBN‐CNCs exhibit increased capacity upon cycling due to enlarged graphitic interlayer spacing. Characterization of graphitic nanostructures confirms that Al doping effectively tailors and enhances their electrochemical properties, providing a new strategy for high‐capacity, fast‐charging graphitic carbon anode materials for next‐generation LIBs.

## Graphical abstract

Graphitic carbon materials are used in lithium‐ion batteries (LIBs) for stability and conductivity, but suffer from low capacity and degradation. This study introduces aluminum (Al) into carbon nanocages (CNCs) via chemical vapor deposition, enhancing performance. Al‐doped CNCs exhibit ultrahigh rate capacity (300 mAh g^−1^ at 10 A g^−1^) and prolonged lifespan, improving lithium‐ion battery efficiency through better structural stability and faster kinetics.

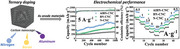

## 1 Introduction

Lithium (Li)‐ion batteries (LIBs) are among the most popular energy storage devices because of their high energy density and excellent charge–discharge reversibility. Therefore, LIBs are widely used in electric vehicles and portable devices.^[^ ^1^, ^2^, ^3^, ^4^ ^]^ At present, commercial LIBs mainly use graphite‐based anodes, which have a low theoretical capacity of 372 mAh g^−1^. Moreover, the capacity of graphitic anodes decreases rapidly during fast charging. This limits the application of graphite‐based LIBs.^[^ ^5^ ^]^ Recently, many anode materials based on Silicon (Si), Lithium (Li), and Phosphorus (P) have been developed because of their high capacities. However, these anodes exhibit poor long‐term stability and cannot meet the requirements of commercial energy storage devices.^[^ ^6^ ^]^ Therefore, the development of fast‐charging graphite‐based anodes with high capacity and stability is regarded as a better strategy. Consequently, extensive research has focused on the design of alternative carbon‐based anode materials using graphene, carbon nanotubes, and carbon nanofibers.^[^ ^7^, ^8^, ^9^, ^10^ ^]^ Heteroatom‐doped carbon materials have been shown to enhance the electrochemical performance of graphitic carbon anodes.^[^ ^11^, ^12^, ^13^, ^14^ ^]^ For example, doping carbon materials with Nitrogen (N), Boron (B), or Sulphur (S) introduces defects and additional active sites for Li‐ion insertion.^[^ ^15^, ^16^, ^17^, ^18^ ^]^ Wu et al. demonstrated that N‐doped (NG) and B‐doped graphene (BG) outperformed undoped graphene as LIB anode materials. NG and BG delivered capacities over 1000 mAh g^−1^ at low currents and maintained 200–235 mAh g^−1^ capacities at high rates up to 25 A g^−1^. Their superior performance was due to their enhanced conductivity, increased number of active sites, and mechanical stability provided by doping.^[^ ^18^ ^]^

Doping carbon materials with two or more heteroatoms can further improve the Li storage performance over homo‐doping.^[^ ^16^, ^17^ ^]^ Synergistic interactions between dual or ternary dopants provide more defects, storage sites, and expanded interlayer spacing.^[^ ^19^, ^20^, ^21^ ^]^ Zhu reported that B and N co‐doped porous graphene (BN‐3DG) exhibited enhanced electrochemical performance. Co‐doping results in a high capacity, superior cycling stability, and excellent rate capability.^[^ ^15^ ^]^ However, despite promising results for doping, few studies have explored the Al doping of carbon nanostructures.^[^ ^22^ ^]^

Carbon nanocages (CNCs), a class of higher‐order carbon structures, have attracted considerable attention owing to their unique structures and properties. CNCs have a hollow or frame‐like structure with small openings/pores in their shells, which consist of nanometer building blocks.^[^ ^23^ ^]^ Owing to their unique hollow structure, high specific surface area, excellent chemical stability, and electrical properties, CNCs have great potential for applications in LIBs, supercapacitors, and other fields.^[^ ^24^ ^]^ Aluminum (Al) doping of LIB cathodes has been shown to improve the cycle and rate performance of batteries.^[^ ^25^ ^]^ The successful doping of Al into carbon nanostructures indicates their good compatibility.^[^ ^26^ ^]^ Therefore, we aimed to dope atomic Al into the CNC structure and apply it to LIB anodes to investigate the electrochemical performance of the doped anodes and the possible synergistic effects.

In this study, we prepared Al, B, and N tri‐doped CNCs (AlBN‐CNCs) via template‐assisted chemical vapor deposition (CVD).^[^ ^27^ ^]^ The ultrahigh specific capacity of CNC materials was attributed to their unique 3D structure, which endowed them with a large electrochemically active surface and mechanical strength. Moreover, the 3D hollow framework provided excellent durability during fast charging. The AlBN‐CNC anode exhibited superior electrochemical performance compared with the N‐doped and BN‐doped CNC anodes. X‐ray diffraction (XRD) and Raman spectroscopy revealed that the AlBN‐CNC anodes possessed a larger interlayer spacing and higher defect density. The specific capacity of the AlBN‐CNC electrode increased to 1200 mAh g^−1^ after 200 cycles at 0.5 A g^−1^ and remained high at 862.82 mAh g^−1^ after 1000 cycles. Its specific capacity was 300.03 mAh g^−1^ at an ultrahigh current density of 10 A g^−1^. Density functional theory (DFT) simulations verified that Al doping sites could reduce Li^+^ insertion energies in graphitic materials. The fast diffusivity of Li ions in the AlBN‐CNC electrode was confirmed by galvanostatic intermittent titration (GITT) and electrochemical impedance spectroscopy (EIS). Ex situ X‐ray diffraction confirmed the enlarged graphitic interlayer spacing of the AlBN‐CNC structures after cycling. In general, the novel AlBN‐CNC electrode enabled higher stability and fast charging capacity because of the lower Li^+^ insertion energies and larger interlayer spacing.

## 2 Results and Discussion

### 2.1 Theoretical Simulations

**Figure 1:**
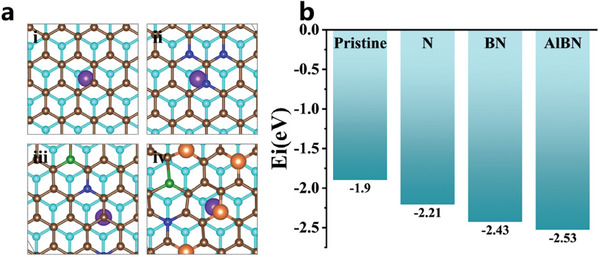
DFT simulations. a) Li intercalation configurations on a‐i) pristine graphene, a‐ii) N‐doped graphene, a‐iii) B–N‐doped graphene, and a‐iv) Al–B–N‐doped graphene. Li, N, B, Al, and upper and lower carbon atoms are represented by the colors purple, blue, green, orange, brown, and cyan, respectively. b) Intercalation energy diagrams of Li ion on different atoms doped graphene configurations.

The intercalation energies of Li^+^ in various types of graphene are calculated using DFT. A bilayer graphene model is employed to evaluate both pristine and doped graphene. The intercalation capacity of Li can be expressed using the following equation:

(1) $$E_{i}=E_{\mathrm{intercalated}}-E_{\mathrm{doped}-G}-E_{\mathrm{bulk}}$$

where *E*_intercalated_ is the total energy of the system after the Li intercalation, *E*_doped−*G*_ is the total energy of the system without Li, and *E*_bulk_ is the chemical potential of Li. *E*_i_ represents the intercalation energy of Li in different systems, including pristine graphene and graphene doped with various atoms. A more negative *E*_i_ value indicates stronger binding energy and easier Li intercalation. **Figure** **1**a illustrates the intercalation configuration of Li in the model, and the corresponding *E*_i_ values are shown in Figure 1b. The intercalation energies for pristine, N‐doped, B–N‐doped, and Al–B–N‐doped graphene are −1.90, −2.21, −2.43, and −2.53 eV, respectively. These results indicate that the intercalation effect of Li is significantly enhanced with increasing amounts of dopants, and that every type of dopant promotes Li intercalation. Moreover, the inclusion of Al further improves the intercalation capability of Li. This improvement is attributed to co‐doping with B and N, as well as the larger atomic size of Al, which affects the overall structure of the system and increases the interlayer spacing.

### 2.2 Material Characterization

**Figure 2:**
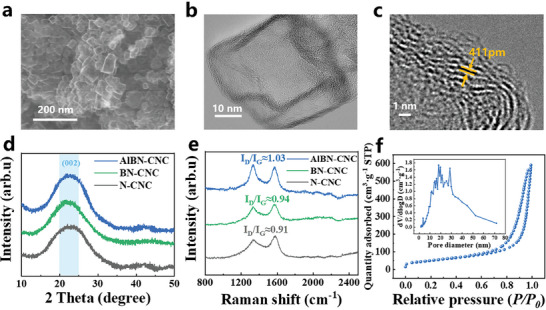
The morphology and structural characterization of CNCs. a) SEM image of AlBN‐CNC. b,c) TEM images of AlBN‐CNC. d) XRD patterns of AlBN‐CNC, BN‐CNC, and N‐CNC. e) Raman spectra of AlBN‐CNC, BN‐CNC, and N‐CNC. f) N_2_ adsorption/desorption isotherm of AlBN‐CNC, with the inset depicting the pore size distribution.

The SEM results (**Figure** **2**a) revealed that the doped CNCs predominantly had irregular hollow cage‐like structures. The morphologies and structures of the BN‐CNCs and N‐CNCs were similar to those of the AlBN‐CNCs (Figures S1 and S2, Supporting Information). Figure 2b,c shows the morphology of the AlBN‐CNCs at different magnifications by TEM imaging. In Figure 2b, the hollow cage structure of the AlBN‐CNCs is clearly visible, confirming that no MgO residue was retained. Figure 2c shows an edge‐shell thickness of 3.95 nm, with a layer spacing of 0.411 nm (as marked in the inset), which exceeds the standard graphite layer spacing of 0.335 nm. The increase in the graphitic layer spacing was primarily due to co‐doping with Al, B, and N heteroatoms. A larger layer spacing facilitates the rapid transport of Li^+^. The morphologies of the BN‐CNCs and N‐CNCs were also characterized using TEM (Figure S3, Supporting Information). Based on the ratio of the shell thickness to the carbon interlayer spacing of the CNCs, the number of carbon layers in the CNCs was estimated to be ≈10. The particle sizes of all CNC samples were concentrated in the 30–50 nm range, which was consistent with the size of the MgO used (30–50 nm), as shown in Figure. S14, Supporting Information.

Figure 2d shows the XRD patterns of AlBN‐CNCs, BN‐CNCs, and N‐CNCs. The broad peaks at 21.64°, 21.74°, and 22.04° correspond to the (002) crystal plane of graphitic carbon, indicating a low degree of graphitization of the material.^[^ ^17^ ^]^ Peak broadening also reflects the degree of disorder in the material.^[^ ^28^ ^]^ Carbon atomic layer spacing was calculated using Bragg's law as follows:

(2) 2dsinθ=nλ

The interlayer spacings of AlBN‐CNCs, BN‐CNCs, and N‐CNCs are 4.1033, 4.0847, and 4.0298 Å, respectively (Table S1, Supporting Information), which further proves that the CNCs have a unique structure with larger interlayer distance than commercial graphite. Al doping results in the largest interlayer spacing for AlBN‐CNCs, followed by BN‐CNCs, suggesting that Al doping can better regulate graphitic carbon nanostructures.

Raman spectroscopy was performed to examine the structural characteristics of the samples. Two prominent peaks at 1344.13 and 1580.77 cm^−^¹ corresponded to the D band (defective feature) and G band (graphitic feature), respectively.^[^ ^29^, ^30^ ^]^ The Raman spectra of the AlBN‐CNCs, BN‐CNCs, and N‐CNCs are shown in Figure 2e, with *I*_D_/*I*_G_ values of 1.03, 0.94, and 0.91, respectively, indicating abundant defects in the CNCs. AlBN‐CNCs exhibited the highest defect density, followed by the BN‐CNCs and N‐CNCs, which was consistent with the XRD results.

The N adsorption–desorption isotherm (Figure 2f) showed a type IV isotherm with a hysteresis loop in the relative pressure range (*P*/*P*_0_) of 0.6–1.0, indicating the presence of numerous pores in the AlBN‐CNC framework. The pore sizes were mainly between 10 and 40 nm, which was consistent with the aforementioned conclusions. The presence of numerous mesopores in the AlBN‐CNC structure can be attributed to its hollow structure and abundance of surface defects.

**Figure 3:**
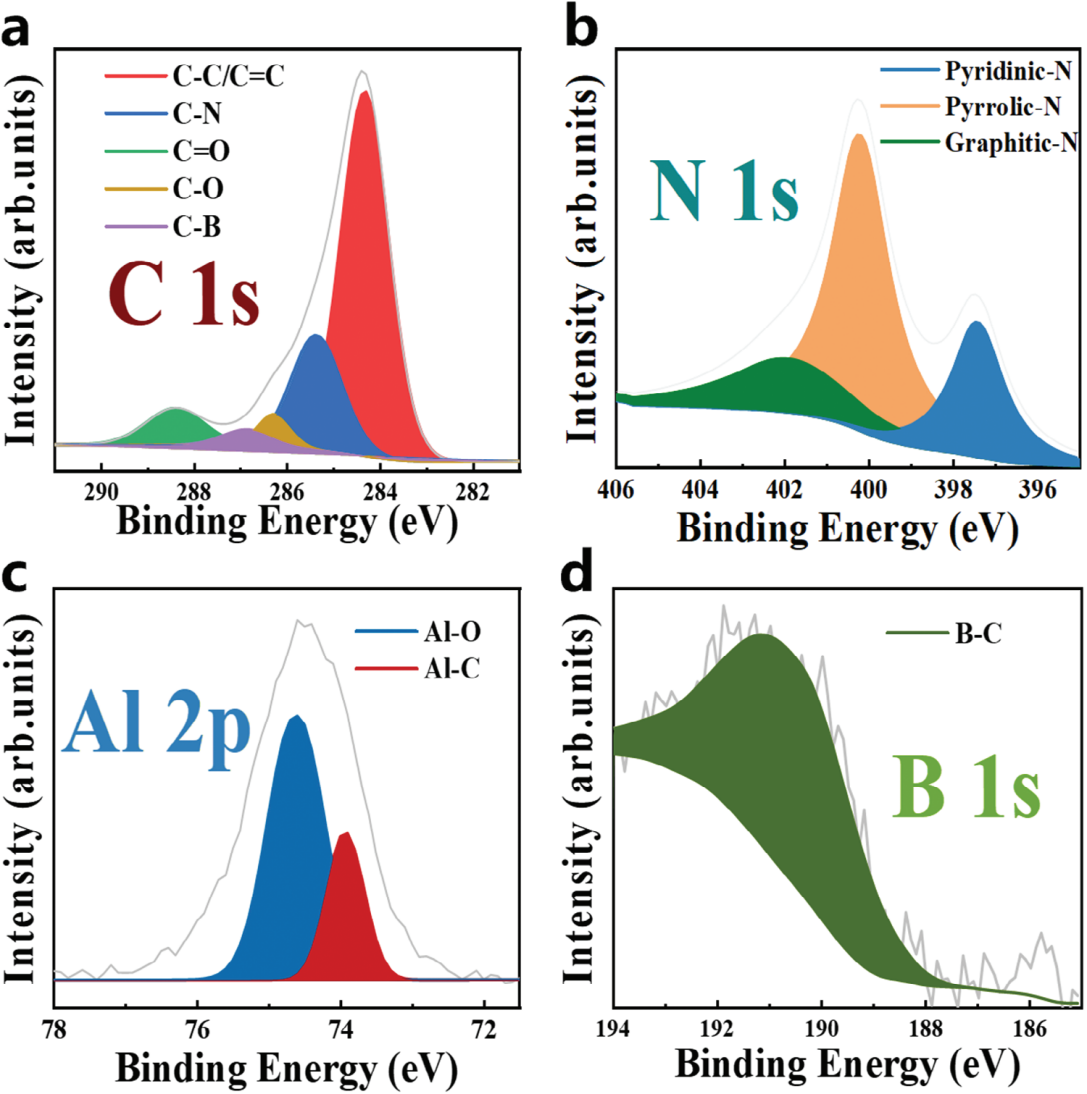
High resolution XPS spectra of AlBN‐CNCs. a) C 1s, b) N 1s, c) Al 2p, and d) B 1s.

XPS was used to analyze the chemical composition and bonding states of AlBN‐CNCs. **Figure** **3**a shows the high‐resolution C 1s spectrum of AlBN‐CNCs, with five distinct peaks corresponding to C─C/C═C (284.3 eV), C─N (285.5 eV), C─B (286.9 eV), C─O (286.3 eV), and C═O (288.4 eV), indicating successful doping with B and N. Figure 3b presents the high‐resolution N 1s spectrum, with peaks at 397.4, 400.2, and 401.9 eV corresponding to pyridinic N, pyrrolic N, and graphitic N, respectively. Figure 3c shows the high‐resolution Al 2p spectrum, which can be deconvoluted into two signals at 73.9 eV (Al─C) and 74.6 eV (Al─O), confirming the successful doping of the CNC framework with Al. The presence of the Al─O bond can be ascribed to inevitable exposure to air. Figure 3d shows the high‐resolution B 1s spectrum, with B primarily bonded as B─C at 190.9 eV. In addition, high‐resolution C 1s, N 1s, and B 1s XPS spectra of BN‐CNCs as well as high‐resolution C 1s and N 1s XPS spectra of N‐CNCs are analyzed (Figures S4 and S5, Supporting Information).

### 2.3 Electrochemical Characterization

**Figure 4:**
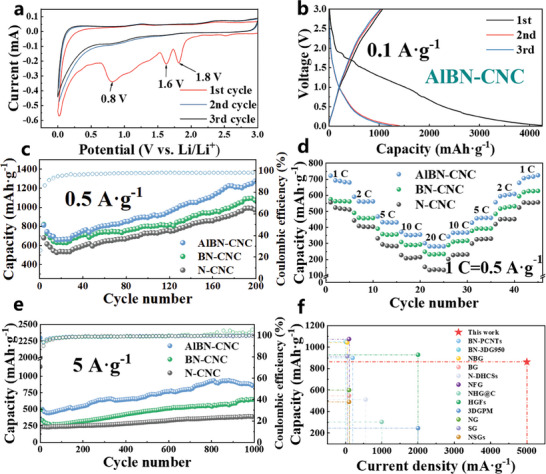
a) Cyclic voltammetry (CV) curves of AlBN‐CNC electrodes during the first three cycles at the scan rate of 0.1 mV s^−1^. b) Galvanostatic charge–discharge profiles of AlBN‐CNCs. c) Cycle performance of N‐CNCs, BN‐CNCs, and AlBN‐CNCs at 0.5 A g^−1^. d) Rate performance of N‐CNCs, BN‐CNCs, and AlBN‐CNCs. e) Long cyclic performance of N‐CNCs, BN‐CNCs, and AlBN‐CNCs at 5 A g^−1^. f) Comparison of the electrochemical performance between the AlBN‐CNC anode and other reported doped‐carbon anodes for LIBs.

Electrochemical tests are conducted using half‐cell configurations. In the CV scans of the AlBN‐CNC electrodes at 0.1 mV s^−1^, three distinct cathodic reduction peaks are observed in the first cycle (**Figure** **4**a). Peaks at 1.8 and 1.6 V correspond to the decomposition of fluoroethylene carbonate (FEC) and vinylene carbonate (VC), respectively, leading to the initial formation of the solid electrolyte interface (SEI) layer.^[^ ^31^, ^32^ ^]^ A significant reduction in the peak at 0.8 V is associated with the decomposition of ethylene carbonate (EC), diethyl carbonate (DEC), and other organic solvents, leading to the formation of a traditional SEI layer.^[^ ^33^, ^34^ ^]^ The sharp cathodic signal between 0.01 and 0.5 V confirms Li^+^ intercalation into the hollow AlBN‐CNC graphitic structure.^[^ ^35^ ^]^ In subsequent cycles, the CV curves are almost identical and overlapping, indicating excellent electrochemical reversibility of the AlBN‐CNC electrodes. The CV curves of BN‐CNCs and N‐CNCs exhibit comparable features (Figure S6, Supporting Information).

At a current density of 0.1 A g^−1^, the AlBN‐CNC electrodes show a remarkable initial discharge capacity of 4271.08 mAh g^−1^ and a charge capacity of 1067.43 mAh g^−1^ (Figure 4b). In contrast, the BN‐CNC electrodes have initial discharge and charge capacities of 4090.15 and 1065.35 mAh g^−1^, respectively (Figure S7a, Supporting Information). The N‐CNC electrodes exhibit a discharge capacity of 3982.64 mAh g^−1^ and a charge capacity of 1041.29 mAh g^−1^ (Figure S7b, Supporting Information). A significant irreversible capacity is observed during the first cycle, leading to a lower initial coulombic efficiency. The low initial coulombic efficiency is due to the large specific surface area of CNCs and numerous surface defects, which consume a large amount of the electrolyte to form an SEI layer.^[^ ^36^ ^]^ The highly consistent curves in the second and third cycles demonstrate good reversibility, a large slope, and no distinct charge–discharge plateaus, reflecting capacitance‐controlled ion storage behavior.^[^ ^37^ ^]^ Figure S8, Supporting Information shows a stair‐discharge image of the AlBN‐CNC electrodes, with no Li dendrite formation observed under SEM.

Figure 4c shows the cycling performance of the AlBN‐CNC electrodes at a current density of 0.5 A g^−1^. The reversible capacity gradually increased from 665.9 mAh g^−1^ at the 15th cycle to 1288.78 mAh g^−1^ at the 200th cycle. In contrast, the reversible capacities of BN‐CNCs and N‐CNCs at the 200th cycle were 964.64 and 1062.31 mAh g^−1^, respectively. Figure 4d compares the rate performance of the AlBN‐CNC, BN‐CNC, and N‐CNC electrodes, where 1 C is defined as 500 mA g^−1^. The rate performance of the AlBN‐CNCs was significantly higher than those of BN‐CNCs and N‐CNCs. At current densities of 1 C, 2 C, 5 C, 10 C, and 20 C, AlBN‐CNCs delivered reversible capacities of 720.85, 590.02, 467.02, 370.92, and 300.03 mAh g^−1^, respectively. When the current density was returned to 0.5 A g^−1^, the capacity recovered to 723.49 mAh g^−1^, consistent with its initial capacity, indicating excellent rate performance and high reversibility. The B and N dopants affected the C configuration and electron transport in AlBN‐CNCs. Further, larger Al atoms cooperated with B and N to create more defects and expand the interlayer graphitic spacing and lithiophilic sites on the CNC surface. These effects contributed to the higher reversible capacity and fast‐charging capability.

From Figure 4e, the specific capacity performances of AlBN‐CNC, BN‐CNC, and N‐CNC electrodes over 1000 cycles at a current density of 5 A g^−1^ can be compared. After 40 cycles, the capacities are 441.82 mAh g^−1^ for AlBN‐CNC, 274.96 mAh g^−1^ for BN‐CNC, and 228.03 mAh g^−1^ for N‐CNC. The capacities gradually increase with further cycling. The capacities of the AlBN‐CNC, BN‐CNC, and N‐CNC electrodes after 200 cycles at different current densities are shown in Figure S9, Supporting Information. After 1000 cycles, the capacities are 862.82 mAh g^−1^ for AlBN‐CNC, 627.64 mAh g^−1^ for BN‐CNC, and 386.82 mAh g^−1^ for N‐CNC at 5 A g^−1^, with capacity retentions of 195.3%, 228.3%, and 169.6%, respectively, demonstrating excellent cycle performance at high current density. This superior performance can be attributed to the nanoporous spherical shell structure of the CNCs, which enhances contact between the electrode material and the electrolyte, thereby increasing the number of electroactive sites.^[^ ^23^ ^]^ The unique hollow, porous carbon layer structure increases the distance between the graphitic layers during charge–discharge cycles, aiding Li‐ion transport, as shown in the ex situ X‐ray diffraction patterns. The Al‐doped AlBN‐CNC electrodes exhibit a much higher capacity than the undoped CNC electrodes, indicating that Al doping significantly improves the capacity.

**Figure 5:**
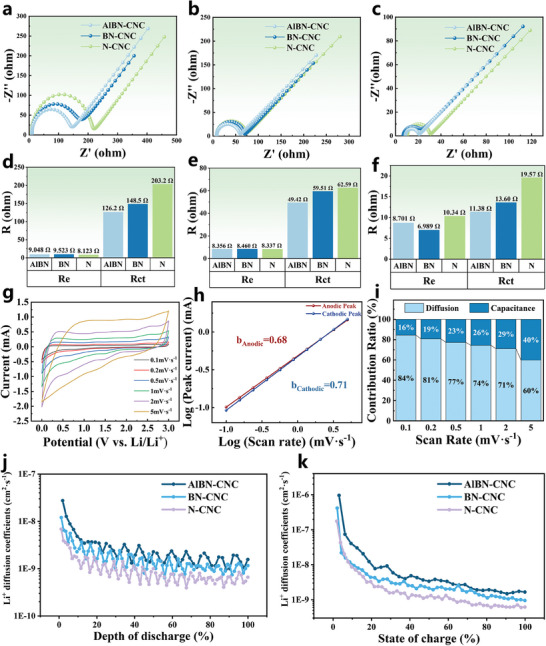
Kinetics analysis of CNC electrodes. a–c) EIS curves of CNC electrodes in initial state, after the first cycle, and after 1000 cycles. d–f) *R*_e_–*R*_ct_ statistics for CNC electrodes under different cycling conditions. g) CV curves of AlBN‐CNC electrodes at 0.1, 0.2, 0.5, 1, 2, and 5 mV s^−1^ scanning rates. h) Plots of log(i)–log(ν) for the *b*‐value determination from CV scans. i) Contribution ratios of capacitance and diffusion processes to capacity at different scanning rates. Diffusion coefficients of Li‐ion batteries during j) lithiation and k) delithiation processes.

In addition, we observe an increasing trend in the discharge capacity of all the samples after repeated discharge cycles at different current densities (Figure 4c,e; Figure S9, Supporting Information). This phenomenon, known as the “activation process,” has been widely documented in studies utilizing metal chalcogenides as anode materials for secondary batteries.^[^ ^38^, ^39^, ^40^ ^]^ The primary reason for the improved cycling performance was that the diffusion kinetics of Li ions were enhanced by the repeated lithiation and delithiation processes. Initially, some active sites, particularly those associated with doped elements, may not have been fully accessible.^[^ ^41^ ^]^ Repeated lithiation and delithiation could induce structural rearrangements or changes in the surface chemistry that could gradually expose or activate these sites, leading to increased capacity. Further, a stable SEI layer was generated and improved cycle performance; the initial SEI formation consumed some Li, but subsequent cycles stabilized the SEI, allowing for more efficient Li‐ion transport and storage.^[^ ^41^, ^42^ ^]^ This can also explain the observed differences in capacities at 5 A g^−1^ (**Figure** **5**d,e). Figure 4f demonstrates that the high current rate capability of AlBN‐CNCs is significantly superior to that of other doped carbon anode materials. A comparison of the rate performance of AlBN‐CNCs with other reported heteroatom co‐doped carbon materials for LIB negative electrodes (Table S2, Supporting Information) reveals that AlBN‐CNCs exhibit markedly better performance.

### 2.4 Kinetics Analysis

Figure 5a–c presents the EIS spectra of CNC electrodes under different cycling conditions: initially (Figure 5a), after the first cycle (Figure 5b), and after 1000 cycles (Figure 5c). The electrical resistance (*R*_e_)–charge transfer resistance (*R*_ct_) statistical plots for these conditions are shown in Figure 5d,e,f, respectively, indicating that the *R*_ct_ of the AlBN‐CNC electrode is lower than that of other CNC electrodes under all cycling conditions. This reduction in *R*_ct_ can be attributed to the increased spacing of carbon layers due to atomic Al‐doping, which facilitates electron and ion transport. As the number of cycles increases, the *R*_ct_ of all three CNC electrodes decreases significantly, which is consistent with the phenomenon of increased capacity. As cycling progresses, the interlayer spacing within the CNCs may undergo slight expansion due to repeated Li‐ion intercalation and deintercalation. This expansion facilitates easier insertion and extraction of Li ions between the carbon layers, thereby reducing the resistance to charge transfer. Further, the decrease in *R*_ct_ values can also be explained by the activation effect that occurs during the initial cycles.^[^ ^41^ ^]^ The repeated cycling can lead to better utilization of the active material and a corresponding reduction in *R*_ct_.

To elucidate the reaction kinetics of the electrodes further, cyclic measurements are performed at various scan rates. Figure 5g shows the cyclic voltammetry curves of the AlBN‐CNC electrodes at scan rates ranging from 0.1 to 5 mV s^−1^. The overall shapes of the curves are similar and depict a more rectangular form at higher scan rates, indicating predominant capacitive behavior. As previously noted, a sharp cathodic signal appears at 0.01–0.5 V, corresponding to the intercalation and deintercalation of Li⁺ within the hollow porous structure of the CNCs. As the scan rate increases, the sharp reduction signal broadens, suggesting a transition from intercalation to capacitive reactions.

To verify the contribution of the capacitive behavior during cycling, the following equation was used to analyze the capacitance effect. The peak current (*i*) and scan rate (*v*) exhibited a power‐law relationship:^[^ ^43^ ^]^

(3) $$i=av^{b}$$

The *b* value was determined from the slope of the log(*i*) versus log(*v*) plot.^[^ ^44^ ^]^ The *b* value reveals the dominant storage mechanism: a value of *b* = 0.5 indicates diffusion control and Faradaic intercalation, whereas a value of *b* = 1 indicates capacitive control owing to the linear relationship between the capacitive current and the scan rate.^[^ ^45^ ^]^ Figure 5h shows the log (*v*) versus log (*i*) plot, from which *b* values of 0.71 and 0.68 for the cathodic and anodic peaks, respectively, are obtained. These *b* values, which are between 0.5 and 1, suggest that the ion storage kinetics of the CNC electrodes are governed by both diffusion and capacitance.

To further distinguish the contributions of capacitive and diffusion‐controlled behaviors to the capacity, the following equation is used:^[^ ^46^ ^]^

(4) $$i=k_{1}\;v+k_{2}v^{1/2}$$

where *v* is the scan rate and *i* is the current at a specific voltage for different scan rates. By fitting the current values at various scan rates and voltage points, the constants *k*_1_ and *k*_2_ are determined, with *k*_1_ *v* and *k*_2_ *v* ^1/2^ corresponding to the capacitive and diffusion‐controlled contributions, respectively.

Figure 5i shows that the capacitance contribution of the AlBN‐CNCs increases from 16% at 0.1 mV s^−1^ to 40% at 5 mV s^−1^. This indicates that, at lower scan rates, the capacity is predominantly diffusion‐controlled, whereas at higher scan rates, the capacitive contribution becomes increasingly significant. The reasons for this phenomenon may be as follows: 1) At higher scan rates, the timescale for the electrochemical reactions becomes shorter. Under these conditions, the electrochemical processes are predominantly controlled by surface reactions rather than by diffusion‐limited processes. As capacitive (non‐Faradaic) processes involve the adsorption and desorption of ions at or near the electrode surface, they can occur much faster than diffusion‐controlled intercalation processes that require ions to penetrate the bulk of the electrode material.^[^ ^47^ ^]^ 2) Diffusion‐controlled processes, which involve ions moving into the electrode material, require more time to occur. As the scan rate increases, the diffusion length decreases, and only the ions near the electrode surface will have sufficient time to participate in the charge storage process. This limitation reduces the contribution from diffusion‐controlled intercalation, further enhancing the relative contribution from capacitive processes.^[^ ^48^ ^]^ 3) The unique porous structure of CNCs provides a large electrochemically active surface area that facilitates rapid ion adsorption and desorption. At higher scan rates, this structure enables significant capacitive charge storage, contributing to the overall capacity. The porous network effectively supports high‐rate surface reactions, leading to an increased capacitive contribution.^[^ ^49^ ^]^

To analyze the dynamic behavior of the electrodes and further explore their kinetics, GITT measurements of the AlBN‐CNC, BN‐CNC, and N‐CNC electrodes were conducted. The pulse current was set to 50 mA g^−1^, with a pulse duration of 30 min and an open‐circuit relaxation time of 5 h. The results are shown in Figure 5j,k. The Li^+^ diffusion coefficient was calculated using the simplified Fick's second law as follows:

(5) $$D=\frac{4}{\pi \tau }\;{\left(\frac{m_{B}V_{M}}{M_{B}S}\right)}^{2}{\left(\frac{\Delta E_{s}}{\Delta E_{\tau }}\right)}^{2}$$

where τ is the duration of the current pulse, Δ*E*_s_ is the quasi‐thermodynamic equilibrium potential difference before and after the current pulse, Δ*E*_τ_ represents the potential difference during the current pulse relaxation, and *m*_B_, *V*_M_, *M*_B_, and *S* are the active mass, molar volume, molar mass, and active surface area of the electrodes, respectively. The calculated $D_{Li^{+}}$ results indicate that the AlBN‐CNC electrodes exhibited higher $D_{Li^{+}}$ values during both the charging and discharging processes than the BN‐CNC and N‐CNC electrodes. This can be attributed to the advanced role of atomic Al doping, which likely accelerated the transport of Li^+^ in the AlBN‐CNC electrodes.

### 2.5 Ex Situ Characterization

**Figure 6:**
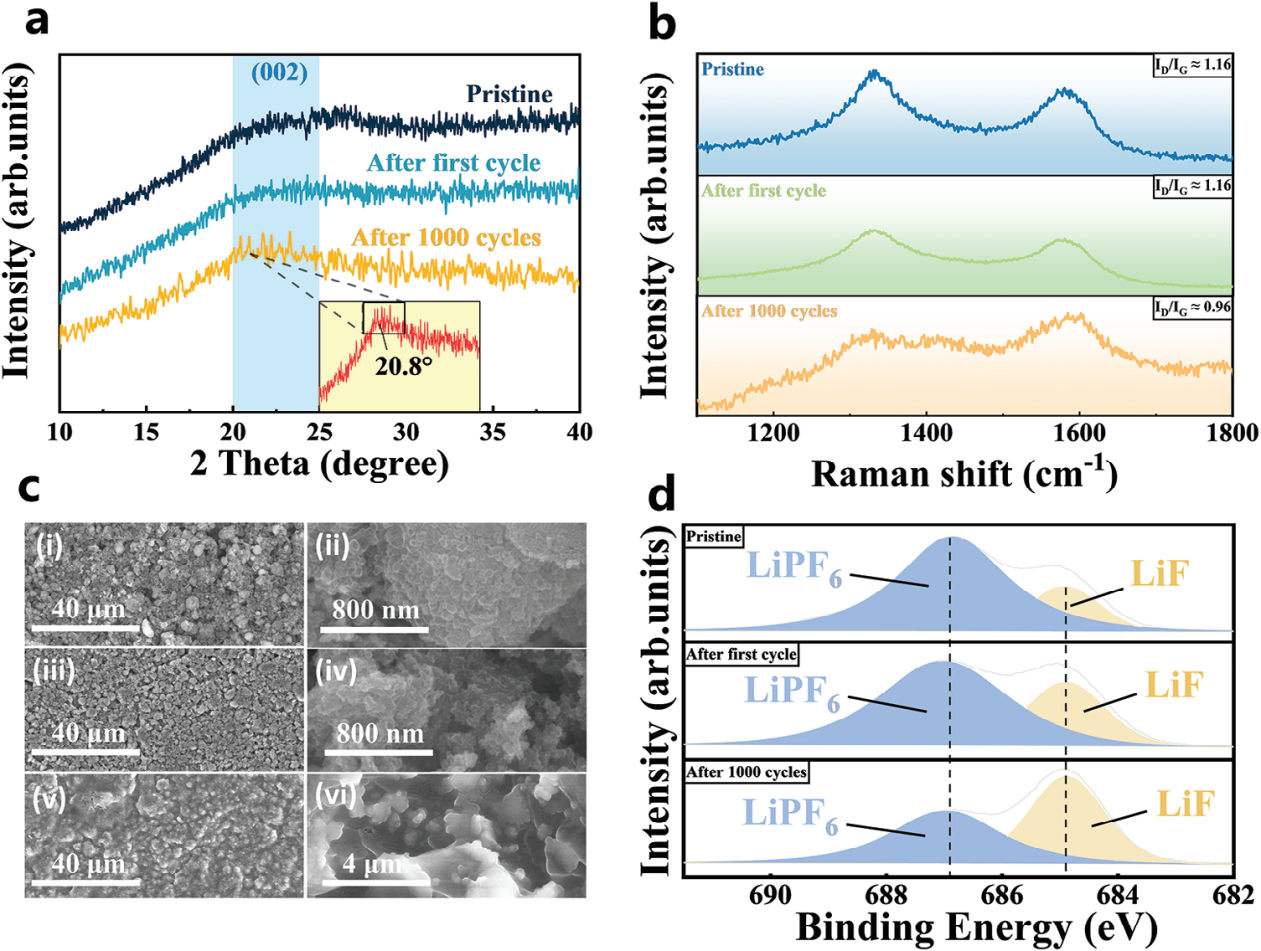
a) Ex situ XRD spectra of AlBN‐CNC electrodes in initial state, after the first cycle, and after 1000 cycles. b) Ex situ Raman spectra of AlBN‐CNC electrodes in initial state, after the first cycle, and after 1000 cycles. c) SEM images of AlBN‐CNC electrodes at low (left) and high (right) magnification at different cycling states. c‐i, ii) Initial state, c‐iii, iv) after the first cycle, and c‐v, vi) After 1000 cycles. d) High resolution F1s spectra of AlBN‐CNC electrodes under different cycling conditions.

To further explain the increase in the capacity of the CNC electrodes during prolonged cycling, ex situ XRD was performed on the CNC electrodes at various cycle stages. The XRD patterns of the AlBN‐CNC electrodes at initial state, after the first cycle, and after 1000 cycles at a current density of 5 A g^−1^ are presented in **Figure** **6**a. A C (002) peak was observed at ≈22° in the XRD patterns of the initial‐ and first‐cycle batteries. After 1000 cycles, the C (002) peak shifted to ≈20.8°. Using the Bragg equation, the interlayer spacing of the AlBN‐CNCs at initial state was calculated as 4.037 Å, which increased to 4.267 Å after 1000 cycles. The graphitic layer expansion is attributed to the rapid insertion and extraction of Li^+^, which in turn boosts the Li‐ion storage capability.^[^ ^50^ ^]^

The AlBN‐CNC electrodes were analyzed ex situ using Raman spectroscopy after different cycles. Figure 6b shows the ex‐situ Raman spectra of the AlBN‐CNC electrodes in their initial state, after the first cycle, and after 1000 cycles. The *I*_D_/*I*_G_ ratio was ≈1.16 in the initial state and after the first cycle, but it decreased to 0.96 after 1000 cycles, indicating a reduction in the surface defect density.

Subsequently, the AlBN‐CNC electrodes were characterized by SEM in the initial state, after the first cycle, and after 1000 cycles to characterize the evolution of the surface morphology. Figure 6c presents ex situ SEM images of the AlBN‐CNC electrodes, with low magnification (a,c,e) on the left and high magnification (b,d,f) on the right. The boundaries of these nanocages blurred as the SEI and Li compounds generated during the cycling penetrated them, causing the surfaces to merge. However, the structural characteristics of the AlBN‐CNCs remained observable. After 1000 cycles, a significant amount of a white substance precipitated on the electrode surface, and no cracks or noticeable volume expansion were observed. Large amounts of Li compounds formed and precipitated on the surface, covering and connecting the AlBN‐CNCs; however, no structural damage was detected. This indicates that the AlBN‐CNCs maintained their mechanical stability even after prolonged cycling.

The F1s XPS spectra of the AlBN‐CNC electrodes under different cycling conditions are shown in Figure 6d. The peak at 686.8 eV can be attributed to residual Li hexafluorophosphate (LiPF_6_) and potential intermediate decomposition products (Li*_x_* PF*_y_*) from electrode demineralization.^[^ ^51^ ^]^ The peak at 684.8 eV is attributed to LiF, the main decomposition product of LiPF_6_.^[^ ^52^ ^]^ As the cycling time increases, the peak intensity of LiF gradually increases, indicating an increase in the amount of LiF at the electrode interface. The C1s XPS spectra of the AlBN‐CNC electrodes at different cycle stages are shown in Figure S12, Supporting Information. The peak at 284.8 eV mainly represents sp^2^ hybridized carbon, and the characteristic peak at 286.7 eV corresponds to C atoms in C─O─C.^[^ ^51^, ^53^, ^54^, ^55^ ^]^ At the initial state and after the first cycle, a weak peak at 290.83 eV represents VC‐containing polymer generated by electrolyte additive decomposition, while the weak peak at 288.7 eV after the first cycle indicates organic alkyl Li carbonate (ROCO_2_Li).^[^ ^52^ ^]^ After 1000 cycles, stronger ROCO_2_Li and Li_2_CO_3_ signals (290.2 eV) are observed. These results indicate that the amounts of LiF and Li_2_CO_3_ at the electrode interface increase significantly upon cycling, which enhances ion transport at the electrode interface.

## 3 Conclusion 

In this study, we successfully synthesized and comprehensively characterized Al‐, N‐, and B‐doped CNCs and measured their electrochemical performances in advanced Li‐ion storage applications. The introduction of Al doping into the CNC structure significantly enhanced the interlayer spacing and surface defects, thereby promoting better Li^+^ diffusion and intercalation. Consequently, AlBN‐CNC electrodes exhibited a high reversible capacity of 862.82 mAh g^−1^ at 5 A g^−1^ after 1000 cycles, showcasing excellent cycle stability and rate performance. Kinetic studies indicated that a combination of diffusion and capacitive control contributed to the superior rate performance of the AlBN‐CNC electrodes. These findings highlight strong potential of the AlBN‐CNC electrodes for practical, high‐performance, and long‐lasting LIBs. Moreover, the role of Al doping was clarified, which is expected to guide further development of high‐capacity graphitic anodes.

## 4 Outlook

The improved performance of AlBN–CNC anodes offers significant potential for future energy storage technologies. Owing to their ultrahigh rate capacity and prolonged cycling stability, AlBN–CNCs are well‐suited for fast‐charging LIBs, particularly in electric vehicles and portable electronics. Compared to traditional graphite anodes, AlBN–CNCs can provide superior rate capability and durability, making them strong candidates for high‐power applications. In addition, AlBN–CNCs may complement or surpass hard carbon in applications such as sodium‐ion batteries, offering a versatile alternative to next‐generation anode materials. The unique properties of AlBN–CNCs, including fast ion diffusion and structural integrity, also open up possibilities for their use in other energy‐storage devices, such as hybrid supercapacitors. Future research should focus on the scalability and commercial integration of these materials, potentially leading to more efficient and sustainable energy‐storage solutions.

## 5 Experimental Section

### Synthesis of AlBN‐CNCs

AlBN‐CNCs were synthesized using atmospheric‐pressure CVD (APCVD) with MgO particles (30–50 nm, 99.9% metal basis, Alab, Shanghai, China) as growth templates. The MgO particles (3 g) were loaded into a quartz boat and placed at the center of the quartz tube of the CVD furnace. Acetonitrile, Al acetylacetonate, and trimethyl borate were mixed in a gas‐washing bottle and a 10% H_2_/90% Ar mixture was used to carry the mixed precursors containing C, N, B, and Al into a CVD furnace at a flow rate of 0.2 L min^−1^. Before heating, the quartz tube was ventilated for 10 min to ensure that the gas was free of impurities. The MgO templates were heated for 180 min at 800 °C at a heating rate of 20 °C min^−1^. In this mixed gas environment, a C shell containing Al, N, and B was deposited layer by layer on the surface of the MgO particles. After heating and when the CVD furnace cooled naturally down to below 200 °C, the quartz boat was taken out and the black MgO particles were transferred into a beaker. The MgO template was then etched with a 1 mol L^−1^ hydrochloric acid solution for 2 h. This step was repeated twice to ensure complete removal of the MgO template. The resulting AlBN‐CNC powders were placed in a small beaker and then freeze‐dried for 48 h to obtain a fluffy AlBN‐CNC solid.

### Electrochemical Measurements

The electrochemical properties of N‐CNC, BN‐CNC, and AlBN‐CNC electrodes were also evaluated. The weight ratio of the active material‐to‐binder‐to‐conductive material in the negative electrode was 7:2:1. After mixing, a specific amount of N‐methyl pyrrolidone (NMP) was added and the mixture was stirred for 5 h. The prepared electrode slurry was coated onto a Cu foil using a scalpel. The coated copper foil was completely dried in an oven at 60 °C. The dried electrode was cut into 14‐mm diameter discs and baked overnight in a vacuum oven at 80 °C to remove any residual moisture. The batteries were assembled in an Ar‐filled glove box with water and oxygen levels less than 0.01 ppm. A 16‐mm diameter Li foil was used as the counter electrode in the assembled half‐cell, and a Celgard 2500 PP membrane was used as the separator. The electrolyte was a mixed solvent composed of 1.0 m LiPF_6_ in EC and 1:1, v/v/v, with 5 wt% FEC and 1 wt% VC additives, purchased from DodoChem Corporation. Long‐term galvanostatic cycling and rate performance tests were conducted using a NEWARE BTS80 programmable battery test system. The potential range was 0.01–3 V versus Li/Li. In order to form a stable SEI layer on the electrode surface and remove excess irreversible capacity, the coin cell was subjected to five pre‐cycles at a current density of 50 mA g^−1^ before the test was performed. EIS and cyclic voltammetry (CV) measurements were performed using a CHI660E electrochemical workstation (CH Instruments, Shanghai). The sampling interval for the CV test was 0.001 V, relaxation time was 2 s, and sensitivity was set automatically. The frequency range for the EIS tests was 10–100 000 Hz. All tests were performed at a temperature of 20 °C.

### Material Characterization

The morphological characteristics of the materials were observed using field‐emission scanning electron microscopy (SEM, Hitachi, SU8010). To further examine the structural details, the materials were characterized using transmission electron microscopy (TEM, FEI, Titan Themis Cubed G2 300) at an acceleration voltage of 80 kV. XRD (Bruker D8 Advance) was used to analyze the phase of the materials at 2*θ* angles between 10° and 80° with Cu Kα radiation (*λ* = 1.5406 Å). The defect densities of the materials were characterized using a Raman spectrometer (Horiba, HR Evolution). The specific surface areas and pore size distributions were analyzed using a Brunauer–Emmett–Teller (BET) specific surface area analyzer (Micromeritics, ASAP 2460). The elemental composition and valence state of the materials were characterized by X‐ray photoelectron spectroscopy (XPS, Escalab 250Xi) using monochromatic Al Kα radiation with a scanning range from 1 to 1500 eV.

### Statistical Analysis

For diameter testing, ≈50 CNC monomers were randomly selected for particle size measurement using Nano Measurer software. Particle size distribution plots were generated to obtain the particle size distribution for each CNC sample.

## Conflict of Interest

The authors declare no conflict of interest.

## Supporting information



Supporting Information

## Data Availability

The data that support the findings of this study are available from the corresponding author upon reasonable request.
