# Supplementary material for: Tailoring the Li+ Intercalation Energy of Carbon Nanocage Anodes Via Atomic Al‐Doping for High‐Performance Lithium‐Ion Batteries
Source: Small. 2024 Oct 2;20(50):2406309. doi: 10.1002/smll.202406309 (PMC11636191; doi:10.1002/smll.202406309)
Supplement: Supplementary file 1 — Supporting Information [file SMLL-20-2406309-s001.docx]

**Tailoring the** **Li^+^ Intercalation Energy of Carbon Nanocage Anodes via Atomic Al-doping for High-performance Lithium-ion Batteries**

*Xingmiao Yu, Jianfei Xiang, Qitao Shi^*^, Luwen Li, Jiaqi Wang, Xiangqi Liu, Cheng Zhang, Zhipeng Wang, Junjin Zhang, Huimin Hu, Alicja Bachmatiuk, Barbara Trzebicka, Jin Chen, Tianxiao Guo, Yanbin Shen^*^, Jinho Choi^*^, Cheng Huang, ^*^ and Mark H. Rümmeli^*^*

**Supplementary Note 1.** GITT measurements.

In this study, the diffusion coefficient of Li^+^ was calculated using the simplified equation of Fick's second law:

$\boldsymbol{D=}\frac{\boldsymbol{4}}{\boldsymbol{\pi\tau}}{\boldsymbol{(}\frac{\boldsymbol{m}_{\boldsymbol{B}}\boldsymbol{V}_{\boldsymbol{M}}}{\boldsymbol{M}_{\boldsymbol{B}}\boldsymbol{S}}\boldsymbol{)}}^{\boldsymbol{2}}{\boldsymbol{(}\frac{\boldsymbol{\Delta}\boldsymbol{E}_{\boldsymbol{s}}}{\boldsymbol{\Delta}\boldsymbol{E}_{\boldsymbol{\tau}}}\boldsymbol{)}}^{\boldsymbol{2}}$ (1)

Where:

$\boldsymbol{\tau}$ is the duration of the current pulse;

$\boldsymbol{\Delta}\boldsymbol{E}_{\boldsymbol{s}}$ is the quasi-thermodynamic equilibrium potential difference before and after the current pulse;

$\boldsymbol{\Delta}\boldsymbol{E}_{\boldsymbol{\tau}}$ represents the potential difference during the current pulse relaxation;

$\boldsymbol{m}_{\boldsymbol{B}}$ is the active mass of the electrode;

$\boldsymbol{V}_{\boldsymbol{M}}$ is the molar volume of the electrode. The molar volume was estimated by considering the combined volumes of carbon and dopant atoms within the nanocage structure, again weighted by their atomic fractions;

$\boldsymbol{M}_{\boldsymbol{B}}$ is the molar mass of the electrode. The molar mass was calculated by taking the weighted average of the molar masses of carbon and the dopant atoms according to their respective atomic fractions in the CNC;

$\boldsymbol{S}$ is the active surface area of the electrode. The active surface area was defined as the total surface area of the carbon nanocages available for lithium-ion interaction, which includes contributions from the doped elements. This was calculated using the Brunauer-Emmett-Teller (BET) method.

**Tables S3, S4, and S5** give the percentage of each element in AlBN-CNC, BN-CNC, and N-CNC measured by XPS, respectively, for the calculation of the molar volume and molar mass of the electrode.

**Table S6** gives the specific surface areas of different CNCs measured using the BET method, which can be used as the active surface area of the electrode.


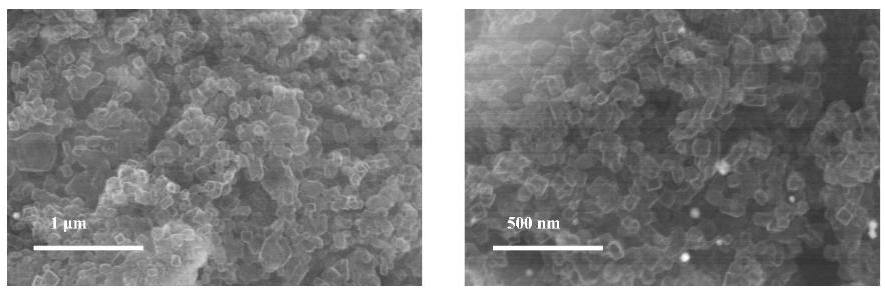


**b**

**a**

**Figure S1.** Low magnification SEM image of AlBN-CNC


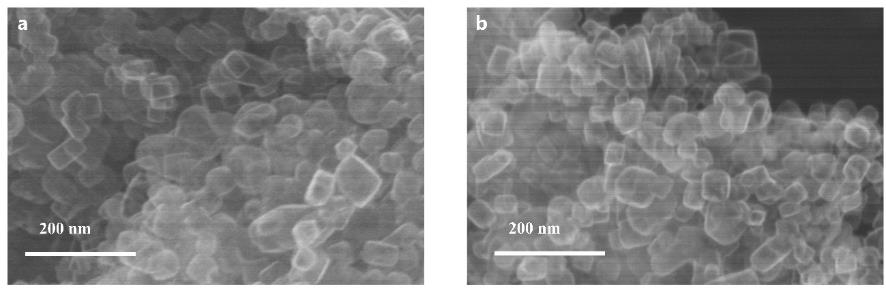


**Figure S2.** SEM images of (a) BN-CNC (b) N-CNC.


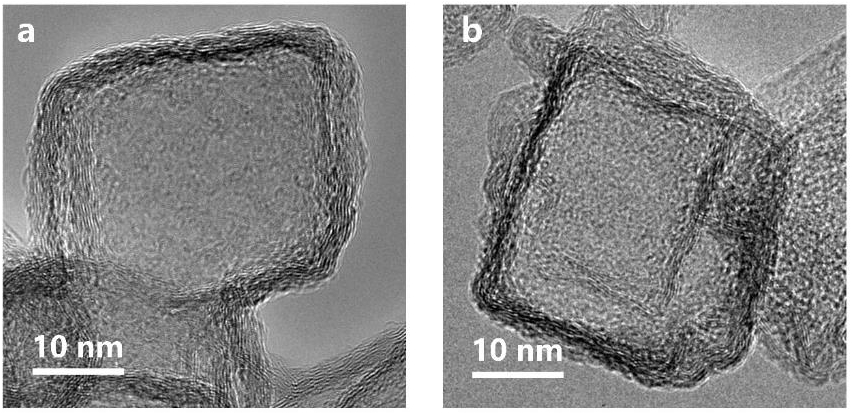


**Figure S3.** TEM images of (a) BN-CNC (b) N-CNC.


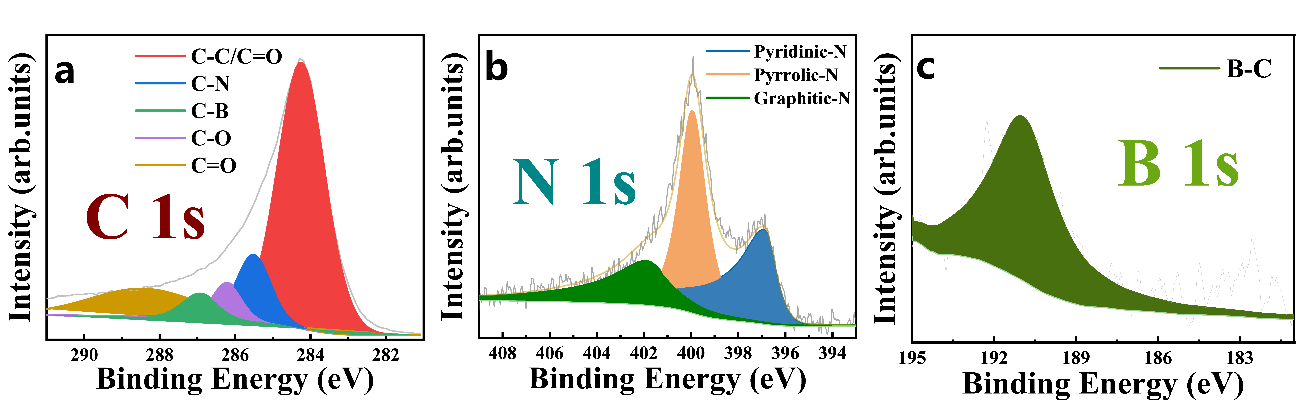


**Figure S4.** High resolution XPS spectrum of BN-CNC. (a) C 1s (b) N 1s (c) B 1s.


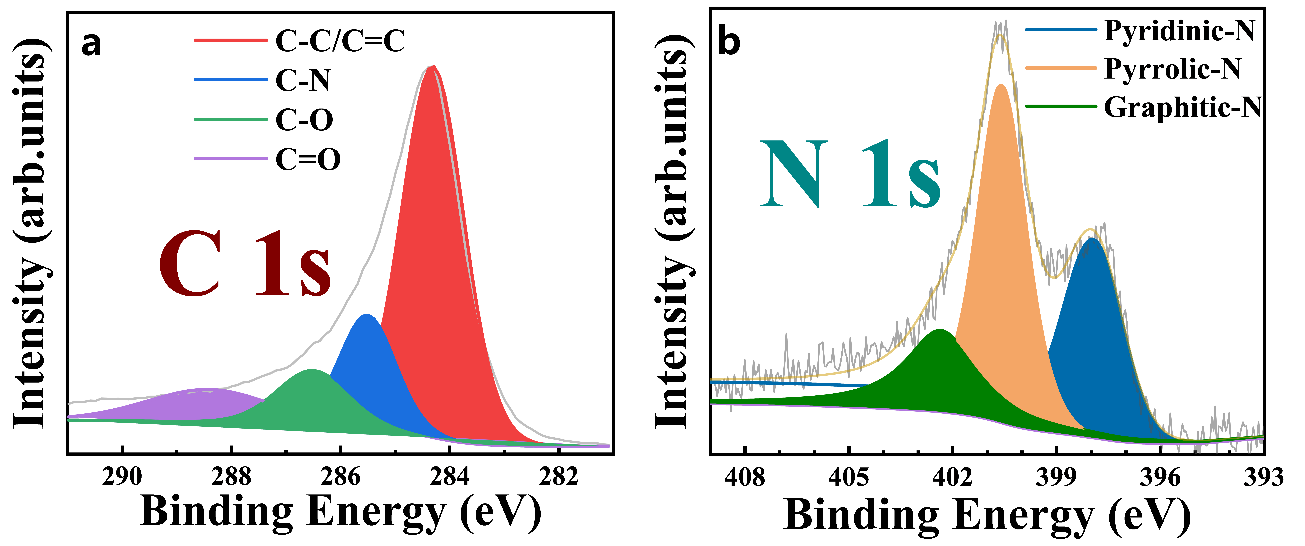


**Figure S5.** High resolution XPS spectrum of N-CNC. (a) C 1s (b) N 1s.


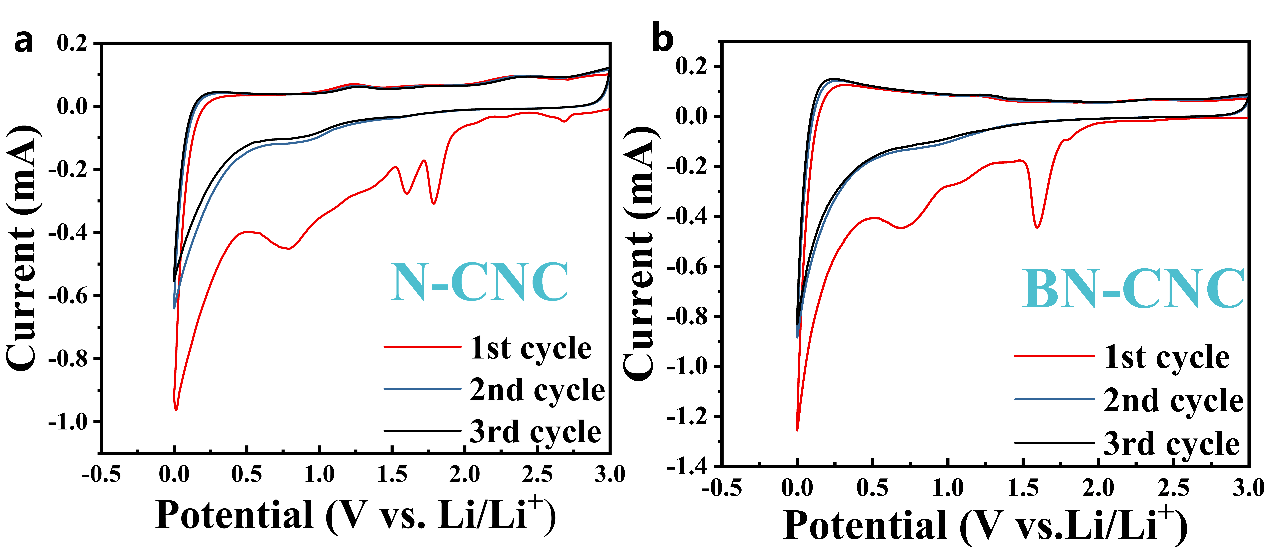


**Figure S6.** CV profiles of (a) N-CNC and (b) BN-CNC electrodes in the first three cycles at a scan rate of 0.1 mV·s^-1^.


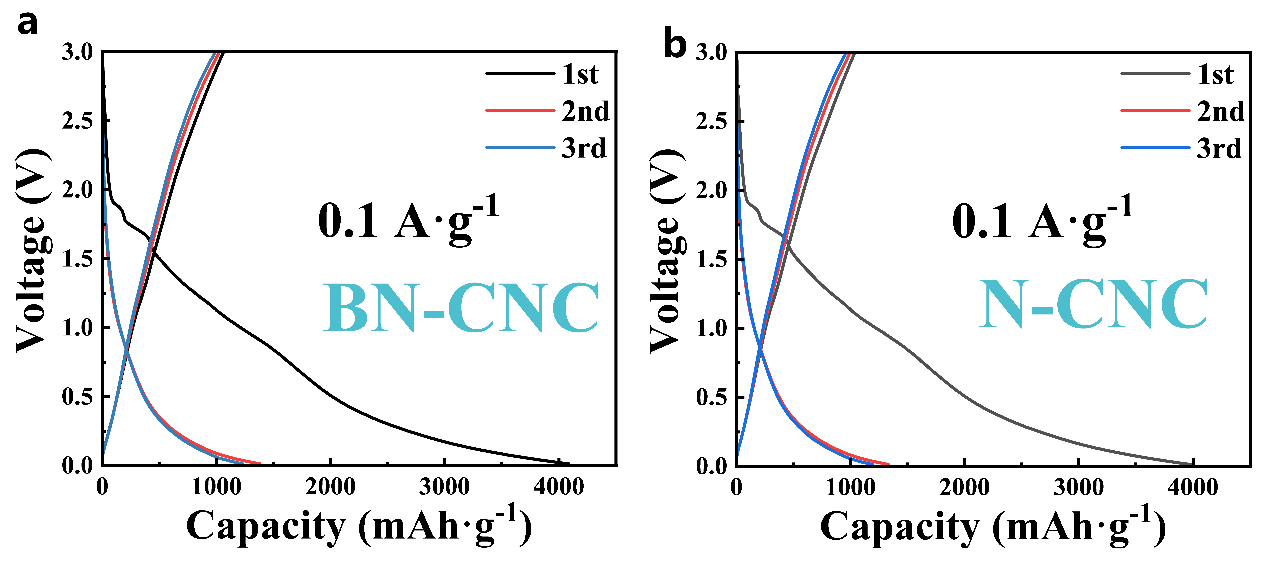


**Figure S7.** Galvanostatic charge-discharge profiles of a) BN-CNC b) N-CNC.


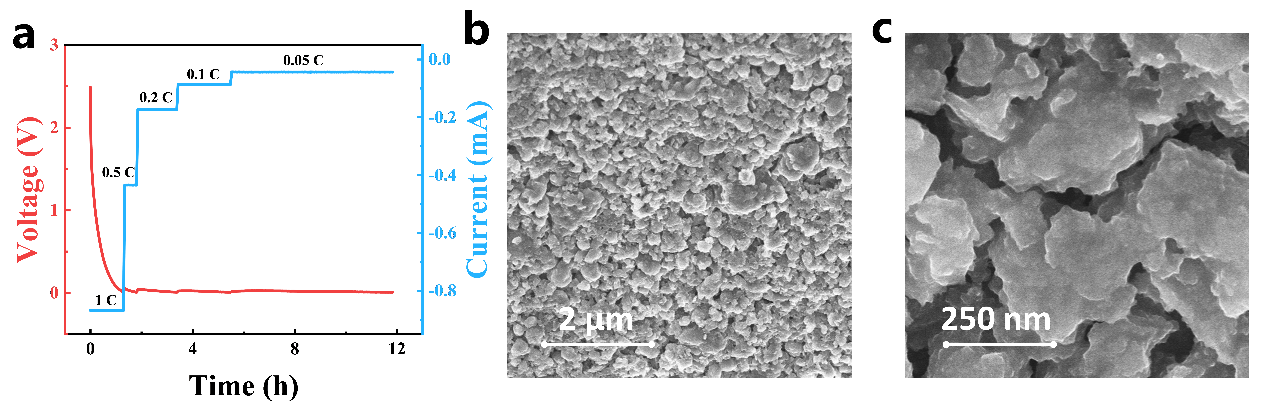


**Figure S8.** (a) Step-discharge image of AlBN-CNC electrode. (b) Low magnification SEM image of AlBN-CNC electrode after step-discharge. (c) High magnification SEM image of AlBN-CNC electrode after step-discharge.


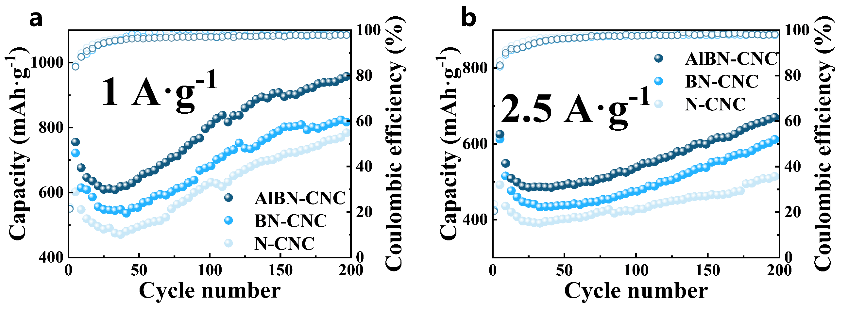


**Figure S9.** Schematic diagram of the capacity of AlBN-CNC, BN-CNC, and N-CNC electrodes after 200 cycles at (a) 1A·g^-1^ (b) 2.5A·g^-1^ (c) 5A·g^-1^.


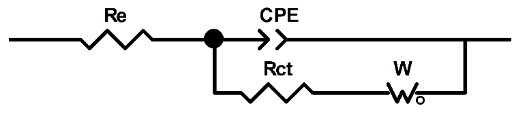


**Figure S10.** Equivalent circuit diagram for electrochemical impedance spectroscopy test of CNC electrodes.


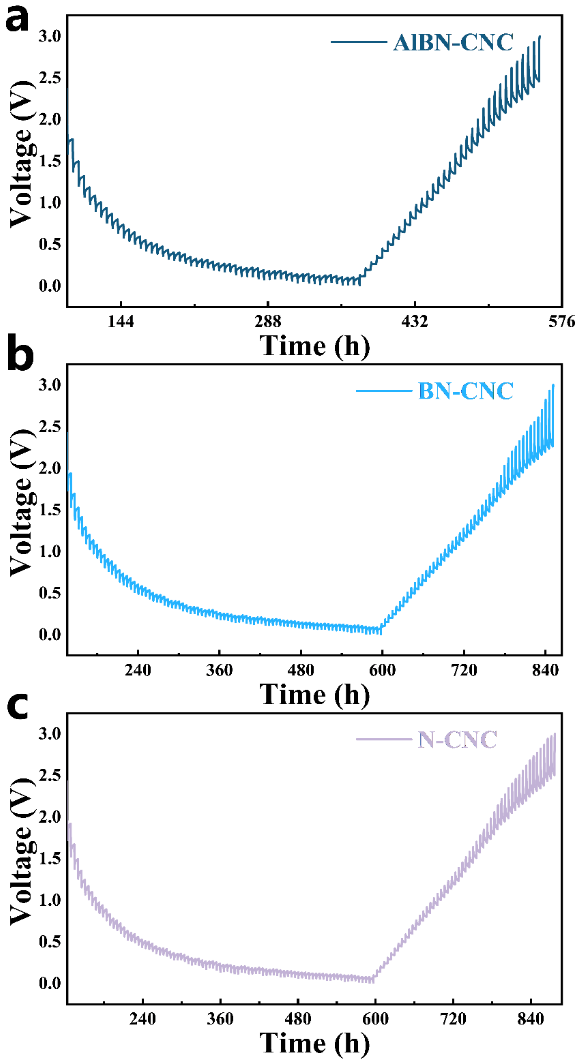


**Figure S11.** GITT profiles for different CNC electrodes: (a) AlBN-CNC. (b) BN-CNC. (c) N-CNC.


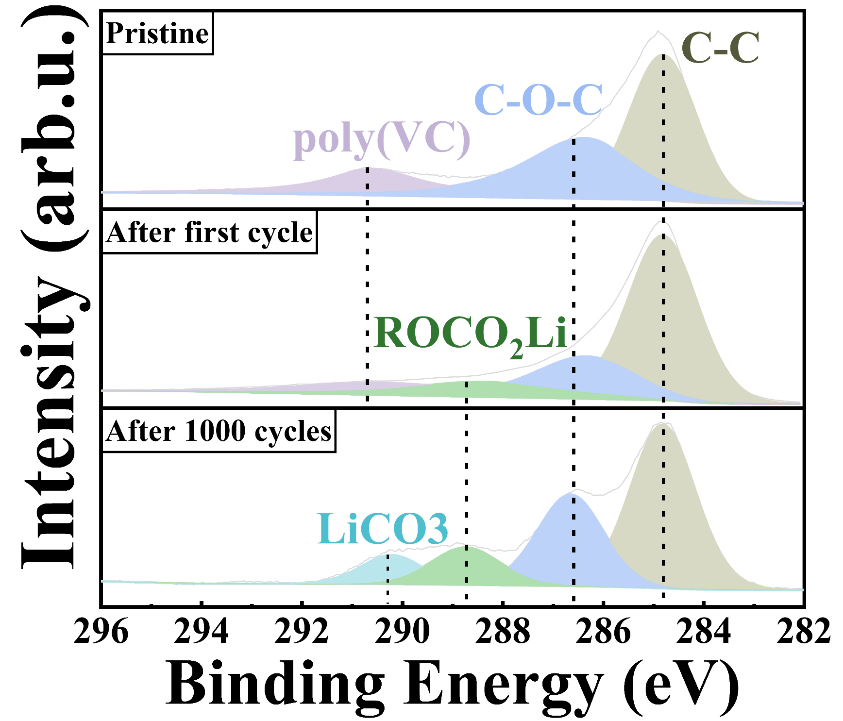


**Figure S12.** The C1s XPS spectra of CNC electrodes at different cycle stages


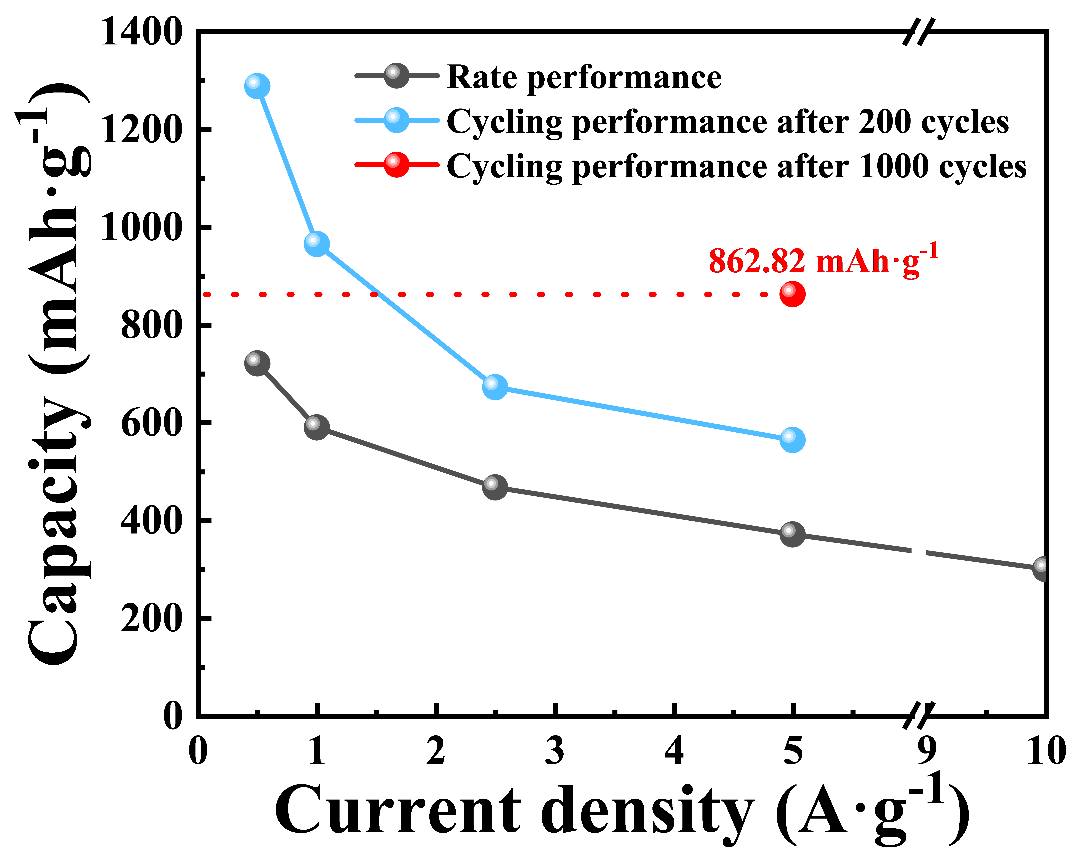


**Figure S13.** Capacity-current density performance of AlBN-CNC at different cycles.


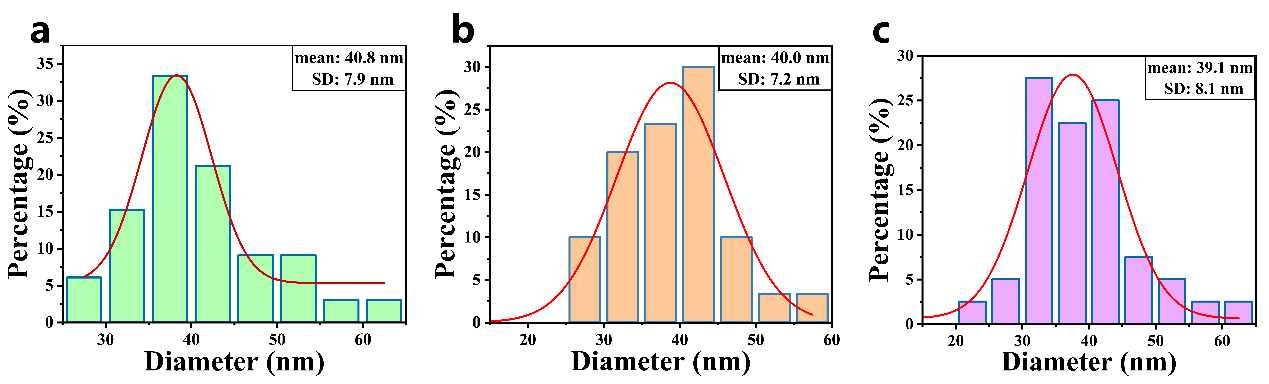


**Figure S14.** Diameter distributions of (a) AlBN-CNC (b) BN-CNC (c) N-CNC.

**Table S1.** The position of (002) peak of different CNCs under XRD and the calculated carbon interlayer spacing.

| **Samples** | **(002) Peak (degree)** | **D_spacings_ (**Å**)** |
| --- | --- | --- |
| **AlBN-CNC** | **21.64** | **4.1033** |
| **BN-CNC** | **21.74** | **4.0847** |
| **N-CNC** | **22.04** | **4.0298** |

**Table S2.** Comparison the electrochemical performance between the AlBN-CNC and other reported doped-carbon anodes for LIB.

| **Electrode** | **Capacity (mAh·g^-1^)** | **Current density (mA·g^-1^)** | **Ref** |
| --- | --- | --- | --- |
| **BN-PCNTs** | **900** | **200** | [1] |
| **BN-3DG950** | **1050** | **10** | [2] |
| **Heteroatom (N, B)-doped chemically derived graphene** | **1043** | **50** | [3] |
| **B-G** | **548** | **100** | [4] |
| **N-DHCSs** | **512** | **558** | [5] |
| **NFG** | **1075** | **100** | [6] |
| **NHG@C** | **304** | **1000** | [7] |
| **HGFs** | **929** | **2000** | [8] |
| **3DGPM** | **245.8** | **2000** | [9] |
| **N-doped graphene** | **600** | **100** | [10] |
| **S-doped graphene** | **915** | **50** | [11] |
| **NSGs** | **490.2** | **100** | [12] |

**Table S3.** Surface content of elements in AlBN-CNC.

| **Elements** | **Content/%** |
| --- | --- |
| **C** | **68.59** |
| **O** | **23.51** |
| **Al** | **4.88** |
| **B** | **0.52** |
| **N** | **2.50%** |

**Table S4.** Surface content of elements in BN-CNC.

| **Elements** | **Content/%** |
| --- | --- |
| **C** | **88.15** |
| **O** | **4.46** |
| **B** | **0.43** |
| **N** | **6.96** |

**Table S5.** Surface content of elements in N-CNC.

| **Elements** | **Content/%** |
| --- | --- |
| **C** | **87.59** |
| **O** | **4.39** |
| **N** | **8.03** |

**Table S6.** BET specific surface area of different samples.

| **Samples** | **Specific surface area** |
| --- | --- |
| **AlBN-CNC** | **935.32 m^2^·g^-1^** |
| **BN-CNC** | **771.60 m^2^·g^-1^** |
| **N-CNC** | **703.64 m^2^·g^-1^** |

**Table S7.** The capacity and cycle number when the cycling capacity of AlBN-CNC reaches peak value under different current densities.

| **Current density (A/g)** | **Maximum capacity (mAh/g)** | **Maximum capacity cycle number** |
| --- | --- | --- |
| **0.5** | **1471.85** | **254th** |
| **1** | **1337.58** | **295th** |
| **2.5** | **871.11** | **374th** |
| **5** | **932.31** | **876th** |

**Table S8.** Comparison of Re and Rct values ​​of AlBN-CNC, BN-CNC and N-CNC in different states.

| **Samples**    **State** | **AlBN-CNC** | | **BN-CNC** | | **N-CNC** | |
| --- | --- | --- | --- | --- | --- | --- |
|  | **Re (Ω)** | **Rct (Ω)** | **Re (Ω)** | **Rct (Ω)** | **Re (Ω)** | **Rct (Ω)** |
| **Initial state** | **9.048** | **126.2** | **9.523** | **148.5** | **8.123** | **203.2** |
| **After first cycle** | **8.356** | **49.42** | **8.460** | **59.51** | **8.337** | **62.59** |
| **After 1000 cycles** | **8.701** | **11.38** | **6.989** | **13.60** | **10.34** | **19.57** |

[1] L. Zhang, G. Xia, Z. Guo, X. Li, D. Sun, X. Yu, *International Journal of Hydrogen Energy* **2016**, 41, 14252.

[2] L. Zhang, Q. He, S. Huang, J. Zhu, J. Key, P. K. Shen, *Inorganic Chemistry Communications* **2018**, 96, 159.

[3] Z.-S. Wu, W. Ren, L. Xu, F. Li, H.-M. Cheng, *ACS nano* **2011**, 5, 5463.

[4] M. Sahoo, K. P. Sreena, B. P. Vinayan, S. Ramaprabhu, *Materials Research Bulletin* **2015**, 61, 383.

[5] K. Zhang, X. Li, J. Liang, Y. Zhu, L. Hu, Q. Cheng, C. Guo, N. Lin, Y. Qian, *Electrochimica Acta* **2015**, 155, 174.

[6] S. Huang, Y. Li, Y. Feng, H. An, P. Long, C. Qin, W. Feng, *Journal of Materials Chemistry A* **2015**, 3, 23095.

[7] X. Yang, C. Zhan, X. Ren, C. Wang, L. Wei, Q. Yu, D. Xu, D. Nan, R. Lv, W. Shen, F. Kang, Z.-H. Huang, *Journal of Solid State Chemistry* **2021**, 303, 122500.

[8] J. Liang, Y. Xu, H. Sun, X. Xu, T. Liu, H. Liu, H. Wang, *Energy Technology* **2020**, 8, 1901002.

[9] B. Zhu, X. Liu, N. Li, C. Yang, T. Ji, K. Yan, H. Chi, X. Zhang, F. Sun, D. Sun, C. Chi, X. Wang, Y. Wang, L. Chen, L. Yao, *Surface and Coatings Technology* **2019**, 360, 232.

[10] Z. Xing, Z. Ju, Y. Zhao, J. Wan, Y. Zhu, Y. Qiang, Y. Qian, *Scientific Reports* **2016**, 6, 26146.

[11] H. Gürsu, Y. Güner, K. B. Dermenci, M. Gençten, U. Savaci, S. Turan, Y. Şahin, *Ionics* **2020**, 26, 4909.

[12] Y. Zhou, Y. Zeng, D. Xu, P. Li, H.-g. Wang, X. Li, Y. Li, Y. Wang, *Electrochimica Acta* **2015**, 184, 24.
